# The Phytochemical, Antifungal, and First Report of the Antiviral Properties of Egyptian *Haplophyllum tuberculatum* Extract

**DOI:** 10.3390/biology9090248

**Published:** 2020-08-25

**Authors:** Ahmed Abdelkhalek, Mohamed Z. M. Salem, Elsayed Hafez, Said I. Behiry, Sameer H. Qari

**Affiliations:** 1 Plant Protection and Biomolecular Diagnosis Department, ALCRI, City of Scientific Research and Technological Applications, New Borg El Arab, Alexandria 21934, Egypt; elsayed_hafez@yahoo.com; 2 Forestry and Wood Technology Department, Faculty of Agriculture (El-Shatby), Alexandria University, Alexandria 21545, Egypt; 3 Agricultural Botany Department, Faculty of Agriculture (Saba Basha), Alexandria University, Alexandria 21531, Egypt; said.behiry@alexu.edu.eg; 4 Biology Department, Al-Jumum University College, Umm Al-Qura University, Mecca 25376, Saudi Arabia; shqari@uqu.edu.sa

**Keywords:** *Haplophyllum tuberculatum*, phytochemical analysis, HPLC analysis, antifungal property, tobacco mosaic virus, antiviral activity

## Abstract

In this study, ethanol whole plant extract (WPE) of *Haplophyllum tuberculatum* was characterized and tested for its antifungal and antiviral activities against *Fusarium culmorum*, *Rhizoctonia solani* and tobacco mosaic virus (TMV). High Performance Liquid Chromatography (HPLC) analysis showed that the main phytochemical constituents of *H. tuberculatum* WPE were resveratrol (5178.58 mg/kg), kaempferol (1735.23 mg/kg), myricetin (561.18 mg/kg), rutin (487.04 mg/kg), quercetin (401.04 mg/kg), and rosmarinic acid (387.33 mg/kg). By increasing *H. tuberculatum* WPE at concentrations of 1%, 2%, and 3%, all of the fungal isolates were suppressed compared to the two positive and negative controls. Under greenhouse conditions, WPE-treated *Chenopodium amaranticolor* plants strongly inhibited TMV infection and significantly reduced TMV accumulation levels when compared to non-treated plants. Moreover, the induction of systemic resistance with significant increases in the transcriptional levels of the pathogenesis-related protein-1 (*PR*-1), chalcone synthase (*CHS*)*,* and hydroxycinnamoyl-CoA quinate transferase (*HQT*) genes for treated plants were noticed at 3 and 5 days post-inoculation (dpi) for both assays. To the best of our knowledge, this is the first reported observation of the antiviral activity of *H. tuberculatum* extract against plant viral infections. Finally, the results obtained suggest that *H. tuberculatum* WPE can be considered a promising source of both antifungal and antiviral substances for practical use and for developing plant-derived compounds for the effective management of plant diseases.

## 1. Introduction

*Haplophyllum* is a genus belonging to the Rutaceae family. It is distributed in different floristic regions. The plant is rich in alkaloids, fixed oils, volatile oils, furanocoumarins, and several classes of compounds such as alkaloids, lignans, coumarins, and flavonoids have been isolated from the aerial parts of *Haplophyllum tuberculatum* [1]. Globally, plant viral diseases constitute severe threats to sustainable development and modern agriculture [2]. Among these viruses, the tobacco mosaic virus (TMV) is one of the most common viral diseases that causes economic losses of and severe damage to quality and crop production worldwide [3,4]. Besides ranking in the top 10 plant viruses in molecular plant pathology, TMV is used as a model virus, and *Chenopodium amaranticolor* as a TMV-local lesion host for different biological studies [5]. Insect-borne viruses such as TMV and cucumber mosaic virus (CMV) can be controlled well by applying induced resistance (IR), even by a biological or chemical inducer [6,7]. In addition, in Egypt, the production of tomato faces the problem of insect-borne viruses, which are very difficult to manage because of their wide host range [8]. Systemic acquired resistance (SAR) is an inducible defense mechanism that plays a central role in disease resistance [9]. Moreover, the chemical induction of SAR treatment induces both pathogenesis-related (PR) protein accumulation and resistance to viruses, bacteria, and the fungus in Arabidopsis plants [10].

Recently, it has been demonstrated that the chemical induction of SAR treatment of vegetable plants protects them against root rot diseases [11]. Several biotic stresses can affect tomato plants more so than other vegetables. Moreover, they are capable of remaining in soil and plant residues for an extended period of time [12]. The soil-borne pathogenic fungus “biotic stresses” can lead to a decrease in crop production of the *Rhizoctonia solani* plant, which causes several injures in the bean of tomato crops [13]. *Fusarium* spp. are an abundant saprophyte in soil and organic matter and are found worldwide. Some strains cause vascular wilt disease in plants, including vegetables, bananas, and date palms such as *Fusarium culmorum*.

Most fungal species, such as the *Fusarium*, *Rhizoctoni*a, and *Penicillium* species, cause mold and discolor wood-based products [14,15]. However, chemical fungicides can induce further problems, harming other living organisms by reduction of useful soil microorganisms [16]. Therefore, alternative methods of pest control could be an approach to reduce the use of pesticides. For instance, biological control strategies are slowly replacing harmful pesticides due to the acceleration of developed biological control products and commercialized forms [17]. Recently, there has been a trend to develop an environmentally safe, long-lasting chemical fungicide based on plant metabolites as an alternative for the control of *Fusarium* diseases, benomyl, and captafol [18,19]. Many plants exhibit antifungal activities that could produce a variety of secondary metabolites against phytopathogenic fungi [20,21,22]. Plant-derived natural products and bioactive compounds include phenols, phenolic acids, quinones, flavones, flavonoids, flavonols, tannins, and coumarins, which are well-known examples for biofungicides [23,24,25,26].

The mycelial radial growth of *Fusarium oxysporum* is reduced by *Azadirachta indica*, *Calotropis procera*, *Citrullus cololcynthis*, *Datura stramonium*, and *Nicotiana tabacum* extracts [27]. Furthermore, *Cinnamomum burmanni* leaf aqueous extract efficiently suppresses the biomass and spore formation of *F. oxysporum f.* sp. *lycopersici* [28]. Meanwhile, the ethanol extracts from *Lowsonia inermis* and *Psidium guajava* are effective in inhibiting the *Fusarium* pathogen [29].

Most approaches applied to control TMV include treating plants with chemical pesticides or using breeding and transgenic plants. However, chemically synthetic pesticides harm the environment and human health; also, transgenic plants have not yet been universally accepted [30]. Therefore, there is still a high demand for discovering more alternative, environmentally friendly, and effective antiviral methods.

Plants are rich sources of bioactive constituents with an antiviral activity that can develop environmentally friendly methods of disease management [31]. Reports of the antiviral activities of plant crude extracts and their constituents against plant viral infection have increased during the last decade [32]. Many plant extracts of *Boerhaavia diffusa*, *Clerodendrum aculeatum*, *Mirabilis jalapa*, *Potentilla arguta*, *Sambucus racemosa*, and *Thuja orientalis* exhibit inhibitory effects against plant viruses [33,34,35,36,37]. Additionally, several virus-inhibiting compounds, including flavonoids, triterpenoids, alkaloids, and proteins, have been isolated from higher plants [31].

*H. tuberculatum* extracts have been noticed to exhibit insecticidal [38], nematicidal [39], antifungal, and antibacterial properties [40,41]. The polyphenolic and alkaloid compounds in the ethyl acetate extract from the leaves of *H. tuberculatum* may be significant contributors to the antioxidant activity of these extracts [42]. Leaf oil extracted from *H. tuberculatum* shows strong anticandidal activity against *Candida krusei* at 30 μg/mL [43]. Also, essential oil has been found to inhibit the growth of *Curvularia lunata* and *F. oxysporum* [40].

The present study aimed to analyze the protective activity and inactivity of the ethanol extract of *H. tuberculatum* against the TMV for the first time. Additionally, the changes in the transcriptional levels of some defense-related genes and TMV accumulation levels at different time intervals were evaluated. Moreover, the antifungal properties of *H. tuberculatum* were assessed against two molecularly identified fungal isolates, namely, *F. culmorum* and *R. solani*.

## 2. Materials and Methods

### 2.1. Preparation of the H. tuberculatum Extract and HPLC Analysis of Phenolic Compounds

Whole plants (WP) of *H. tuberculatum* collected from the northwest of Egypt in April 2018 were air-dried at room temperature for one week. The dried WP was ground to a fine powder using a small laboratory mill. Approximately 100 g of the powdered WP of *H. tuberculatum* was extracted by the soaking method [44] with 200 mL of ethanol solvent for three days. After the extraction process was finished, the extract was filtered through a cotton plug and then with Whatman No. 1 filter paper. The filtered extract was concentrated by evaporating the ethanol solvent to obtain the *H. tuberculatum* whole plant extract (WPE). To prepare the concentration of the extract, the *H. tuberculatum* WPE was dissolved in dimethyl sulfoxide (10% DMSO = 10 mL DMSO (99.999%) + 90 mL distilled water), and the concentrations levels of 1%, 2%, and 3% were obtained. Then, 1, 2, and 3 g from the extract were dissolved in 100 mL of 10% DMSO to obtain the extract concentrations of 1%, 2%, and 3%, respectively. The extract was analyzed for its polyphenolic compounds using Agilent 1260 Infinity HPLC Series (Agilent, Santa Clara, CA, USA), equipped with a Quaternary pump and a Zorbax Eclipse Plus C18 column (100 × 4.6 mm i.d.) in *H. tuberculatum* WPE [15,44,45,46,47,48]. The analysis was carried out based on the 23 standard phenolic compounds [47].

### 2.2. Antifungal Property of Wood Treated with H. tuberculatum WPE

*F. culmorum* and *R. solani* as common molds were used for the antifungal bioassay previously isolated from twigs, trunks, and roots were collected from sweet orange trees showing cankers, dry root rot, wilt and decline at Bader district, Egypt. The isolated fungal colonies were characterized morphologically and molecularly by the internal transcribed spacer region of the rDNA (ITS) gene and the amplified fragments were sequenced and the generated sequences were deposited in Genbank under accession numbers MH352452 and MH352450, respectively [49,50]. Briefly, *Melia azedarach* wood samples were air-dried and prepared with an approximate dimension of 0.5 × 1 × 1 cm. The prepared wood samples were autoclaved for 20 min at 121 °C, and then left to cool. Three wood samples were used for each concentration for each fungus, as well as for the positive (25 µg of fluconazole) and negative (10% DMSO) controls. The antifungal activity of the wood-treated extract in terms of the inhibition percentage of fungal linear growth (IPFLG) was measured following our previous work [15,49,50,51], using the following formula; IPFLG (%) = [(G_C_ − G_T_)/G_C_] × 100, where G_C_ and G_T_ represent the average diameters of the fungal colony of control and treatment, respectively.

### 2.3. Source of Virus, Inoculum Preparation and Antiviral Activity Assays

Egyptian TMV strain KH1 (Acc# MG264131) was propagated in *N. tabacum* and purified as previously described [52]. Then, 50 μL of 20 µg/mL TMV, diluted with 0.1 M phosphate buffer, pH 7.2, was used as the viral inoculum. The prepared concentration of the WPE of *H. tuberculatum* (200 µg/mL) diluted with sterile distilled H_2_O was prepared from a stock solution of 2% of *H. tuberculatum* WPE dissolved in DMSO. A mixture of equal volumes of DMSO and sterile distilled H_2_O was used as the negative control. By using the half-leaf method [47,53], with *C. amaranticolor* as a TMV-local lesion host, the assessment of the antiviral activity of WPE was evaluated according to the inhibition percentage toward number of local lesions. The inhibitory effects were calculated according to the following formula: [I = (1 − T/C) × 100], where I is the inhibition effect, T is the number of local lesions on the treated halves of the leaves, and C is the number of local lesions on the non-treated halves of the leaves.

### 2.4. Protective and Inactivity of WPE Assays

Under greenhouse-controlled conditions, *C. amaranticolor* seeds were surface sterilized and sown in plastic pots (20 cm in diameter) filled with sterilized soil. At the 5–6th leaf stage, plants were subjected to two assays, and each assay had three treatments replicated three times.

In the protective assay, the upper-right halves of the leaves were treated with *H. tuberculatum* WPE 24 h before mechanical viral inoculation. In contrast, the upper-left halves of the leaves were inoculated with TMV only without any treatment [54,55]. Mock leaves treated with a mixture of equal volumes of DMSO, sterile distilled H_2_O, and phosphate buffer with carborundum were used as the controls. The local lesion development numbers were recorded at 3–5 days post-inoculation (dpi).

In the inactivity assay, the upper-right halves of the leaves were mechanically inoculated with an *H. tuberculatum* WPE–TMV mixture, in which an equal volume of *H. tuberculatum* WPE was mixed with the same amount of purified TMV and incubated for 1 h. In contrast, the upper-left halves of the leaves were mechanically inoculated with TMV only without any treatment. The observed number of local lesions was recorded 4–5 dpi.

### 2.5. Plant Total RNA Extraction and cDNA Synthesis

Total RNA was extracted from the *C. amaranticolor* halve of the leaves (0.1 g fresh weight), which were collected at 3 and 5 dpi using the RNeasy plant mini kit according to the manufacturer’s instructions (QIAGEN, Hilden, Germany). After treatment with RNase-free DNase to eliminate genomic DNA, the concentration and quality of the extracted RNA were determined at *A*260/*A*280 and *A*260/*A*230 using SPECTROstar Nano (BMG Labtech, Ortenberg, Germany). In contrast, the integrity of the RNA was assessed by the agarose gel electrophoresis technique [56]. First-strand cDNA was synthesized using 1 μg of total RNA with random oligohexamers and oligo (dT) primers, as described previously [57]. Then, RT-PCR was performed in two steps: 42 °C for 1 h and then 72 °C for 10 min. The reaction mixture was stored at −20 °C until used.

### 2.6. Quantitative Real-Time PCR (qPCR) Assay and Data Analysis

The effects of *H. tuberculatum* WPE on the expression of the accumulation levels of the TMV and *C. amaranticolor* defense system were studied using the qPCR technique. Different primer sets (Table 1) specific to pathogenesis-related protein-1 (PR-1), chalcone synthase (CHS), hydroxycinnamoyl-CoA quinate transferase (HQT), and TMV coat protein (CP) genes were used in this study. The housekeeping gene *β*-actin (Table 1) was used as a reference gene for the normalization of the transcript expression levels. The qPCR efficiency was determined for each gene and was between 93% and 100% for all genes. Each sample in all reactions was run in triplicate on a Rotor-Gene 6000 (QIAGEN, ABI System, Hilden, Germany) using the SYBR Green PCR Master Mix (Fermentas, Waltham, MA, USA) [58]. The single and discrete peak of the melting curve analysis at 55–95 °C confirmed the single amplified product for all genes. The amplification programs and the relative expression ratios were accurately quantified and calculated, as described previously [59,60]. Relative expression levels of more than 1 demonstrate an increase in accumulation (i.e., up-regulation), while values lower than 1 show a decrease in expression (i.e., down-regulation).

### 2.7. Statistical Analysis

The relative expression levels of the antivirus activity data were analysed by one-way analysis of variance (ANOVA) using CoStat software. At the same time, significant differences were determined according to the least significant difference (LSD) *p* ≤ 0.05 level of probability, and the standard deviation (SD) is shown as a column bar. Compared to the controls, relative expression levels higher than 1 demonstrated an increase in gene expression (i.e., up-regulation), while values lower than 1 showed a decrease in expression levels (i.e., down-regulation). Data of the antifungal property (i.e., the inhibition percentage of fungal linear growth) as affected by the tested concentrations (1%, 2%, and 3%) compared to the positive and negative controls were statistically analyzed using one-way ANOVA and processed with the Statistical Analysis Software (SAS) system [65]. The differences among the mean of the treatments were recorded using LSD_0.05_.

## 3. Results

### 3.1. Polyphenolic Compounds in the Ethanol Extract

Table 2 shows the polyphenolic compounds found in the ethanolic WPE of *H. tuberculatum*. The main polyphenolic compounds were resveratrol (5178.58 mg/kg), kaempferol (1735.23 mg/kg), myricetin (561.18 mg/kg), rutin (487.04 mg/kg), quercetin (401.04 mg/kg), and rosmarinic acid (387.33 mg/kg).

### 3.2. Antifungal Property

**Figure 1:**
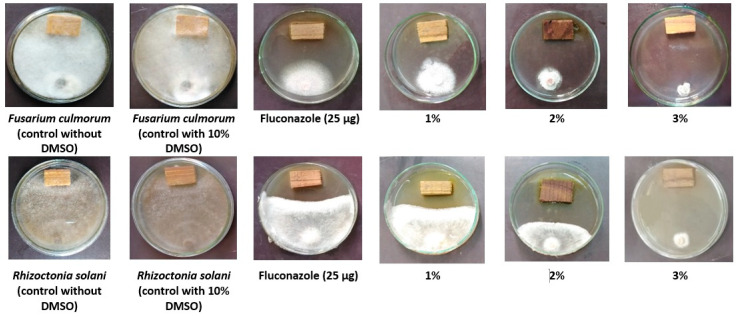
Visual observation of the antifungal property of *Haplophyllum tuberculatum* whole plant extract (WPE) against *Fusarium culmorum* and *Rhizoctonia solani*.

Figure 1 shows the inoculated wood treated with the tested concentrations (1%, 2%, and 3%) prepared from the *H. tuberculatum* WPE with the two fungi *F. culmorum* and *R. solani*. It can be seen from the Petri dishes that with an increase in the extract concentration from 1% to 3%, fungal linear growth was suppressed. In addition, the positive control (25 µg of fluconazole) showed some inhibition in the growth of the tested fungi, while complete growth was recorded in the negative control (10% DMSO). The results of the inhibition percentage of fungal linear growth (IPFLG) are presented in Table 3. *H. tuberculatum* WPE (3%) followed by *H. tuberculatum* WPE (2%) showed the highest IPFLGs of 82.96% and 72.96%, respectively, against *F. culmorum* and were higher than 25 µg of fluconazole (53.70%). *H. tuberculatum* WPE at 3%, 2%, and 1% showed the highest IPFLGs against *R. solani* with values of 93.70%, 66.29%, and 49.62%, respectively, which were more elevated than the value from 25 µg of fluconazole (42.96%).

### 3.3. Effect of H. tuberculatum WPE on Disease Severity and TMV Accumulation Levels

**Figure 2:**
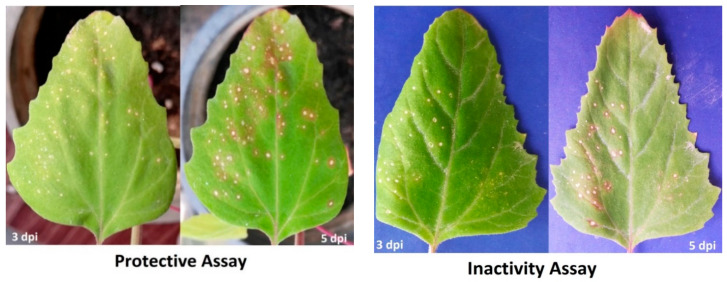
A photograph showing the disease symptoms on *Chenopodium amaranticolor* leaves infected with tobacco mosaic virus (TMV) at 3 and 5 days post-inoculation (dpi) of the protective activity and inactivity of *H. tuberculatum* whole plant extract (WPE) (200 μg/mL). The left-hand sides of the leaves were inoculated with TMV without any treatment, while the right-hand sides of the leaves were treated with WPE.

Under greenhouse conditions, the application of *H. tuberculatum* WPE (200 µg/mL) to *C. amaranticolor* plants significantly reduced the disease severity and decreased the TMV accumulation levels when compared to non-treated plants. The inhibitory effects of *H. tuberculatum* WPE were calculated by comparing the number of developed local lesions on the inoculated leaves at 5 dpi. In the protective assay, the calculated numbers of the local lesions on treated leaves (24 h before virus challenge) were significantly lower than that on non-treated leaves (Figure 2).

**Figure 3:**
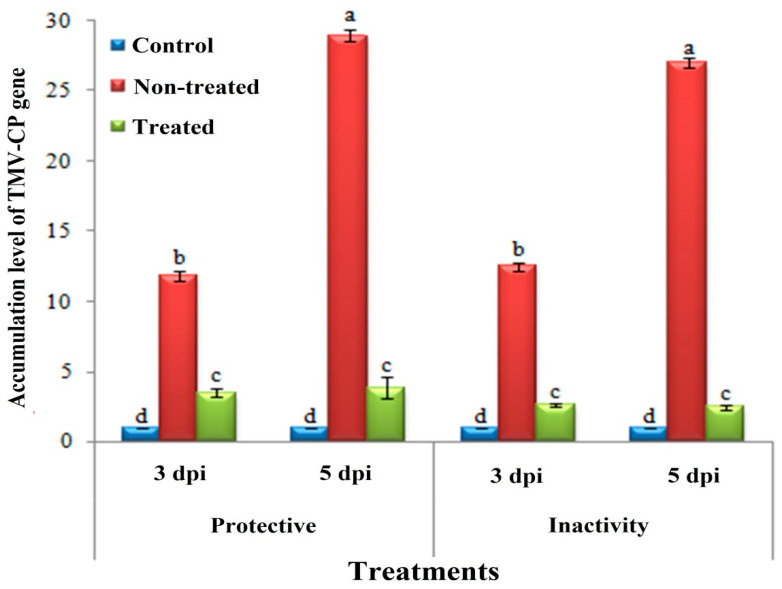
A histogram showing the accumulation levels of the TMV-CP gene at 3 and 5 days dpi with the protective activity and inactivity of *H. tuberculatum* WPE treatments (200 μg/mL). Control = mock-treated plants; non-treated = plants inoculated with TMV only without any treatment; treated = plants treated with WPE, 24 h before inoculation of TMV for the protective assay and 24 h after inoculation of TMV for the inactivity assay. Columns represent a mean value from three biological replicates and the bars indicate the standard deviation (SD). Significant differences between samples were determined by one-way analysis of variance (ANOVA) using CoStat software. Means were separated by the least significant difference (LSD) test at *p* ≤ 0.05 and indicated by lowercase letters. Columns with the same letter do not differ significantly.

Moreover, the *H. tuberculatum* WPE showed an inhibitory effect of 65.38 ± 2.4%. On the other hand, the inactivity assay showed a higher inhibitory effect against TMV infection, with an inhibition rate of 95.73 ± 1.2% (Figure 3). No symptoms were observed on the mock-treated plants. Meanwhile, by using a specific primer of TMV-CP, the level of TMV-CP transcripts significantly decreased in *H. tuberculatum* WPE-treated plant tissues when compared to non-treated tissues. Compared to mock tissues at 5 dpi, the non-treated tissues showed higher accumulation levels of TMV with relative accumulation levels of 28.918- and 27.042-fold change for the protective activity and inactivity treatments, respectively. Notably, *H. tuberculatum* WPE-treated tissues exhibited a considerably decreased TMV concentration level. Compared to the controls, the inactivity and protective activity treatments showed TMV accumulation levels of 2.470- and 3.499-fold change, respectively (Figure 3).

### 3.4. Protective Assay: Changes in the Transcriptional Levels of PR-1, CHS, and HQT

**Figure 4:**
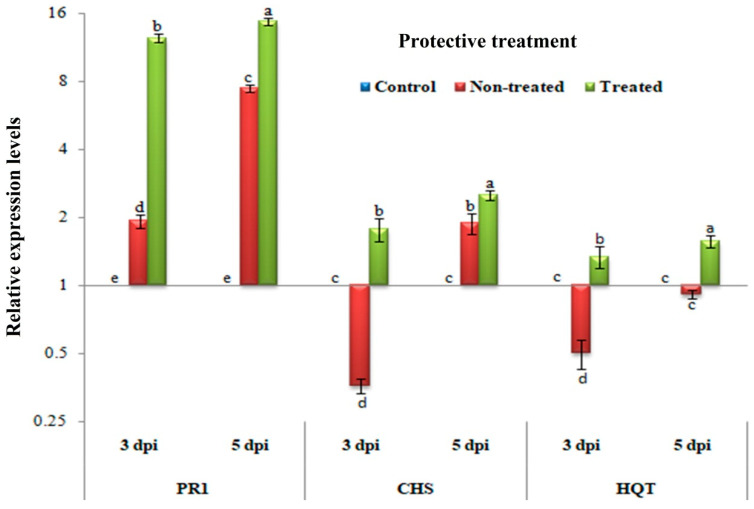
A histogram showing the relative expression levels of the PR-1, CHS, and HQT genes at 3 and 5 dpi of *H. tuberculatum* WPE treatments (200 μg/mL) in the protective activity assay. Control = mock-treated plants; non-treated = plants inoculated with TMV only without any treatment; treated = plants treated with *H. tuberculatum* WPE, 24 h before inoculation of TMV for the protective assay and 24 h after inoculation of TMV for the inactivity assay. Columns represent the mean value from three biological replicates and the bars indicate SD. Significant differences between samples were determined by one-way ANOVA using CoStat software. Means were separated by the LSD test at *p* ≤ 0.05 and indicated by lowercase letters. Columns with the same letter do not differ significantly.

Figure 4 shows significant increases in the relative expression levels of PR-1, CHS, and HQT in plants treated with *H. tuberculatum* WPE when compared to that in non-treated plants (*p* ≤ 0.05) at 3 and 5 dpi. Compared to the controls, a significant up-regulation of PR-1 with relative expressions of 1.926- and 7.467-fold change were observed in non-treated tissues at 3 and 5 dpi, respectively. However, *H. tuberculatum* WPE-treated tissues exhibited overexpression of PR-1 with relative expression levels of 12.436- and 14.750-fold change at 3 and 5 dpi, respectively, compared to the controls. For the CHS transcripts, at 3 dpi, up-regulation with a significant relative expression level of 1.778-fold change was observed in *H. tuberculatum* WPE-treated tissues. In contrast, down-regulation with a relative expression level of 0.359-fold change was observed in non-treated tissues when compared to the control tissues. On the other hand, up-regulation with relative expression levels of 1.880- and 2.512-fold change were showed in non-treated and *H. tuberculatum* WPE-treated tissues, respectively, at 5 dpi compared to the controls. Concerning the HQT gene, significant up-regulation with relative expression levels of 1.340- and 1.573-fold change were shown only in *H. tuberculatum* WPE-treated tissues at 3 and 5 dpi, respectively, when compared to the controls. The down-regulation of HQT with relative transcriptional levels of 0.502- and 0.913-fold change lower than the controls was observed in non-treated tissues at 3 and 5 dpi, respectively. Consequently, treatment of *C. amaranticolor* tissues with *H. tuberculatum* WPE 24 h before TMV challenge induced the expression of HQT. However, TMV induced the expression of PR-1 and CHS, while the *H. tuberculatum* WPE applications triggered the expression of both genes at 3 and 5 dpi.

### 3.5. Inactivity Assay: Changes in Transcriptional Levels of PR-1, CHS, and HQT

**Figure 5:**
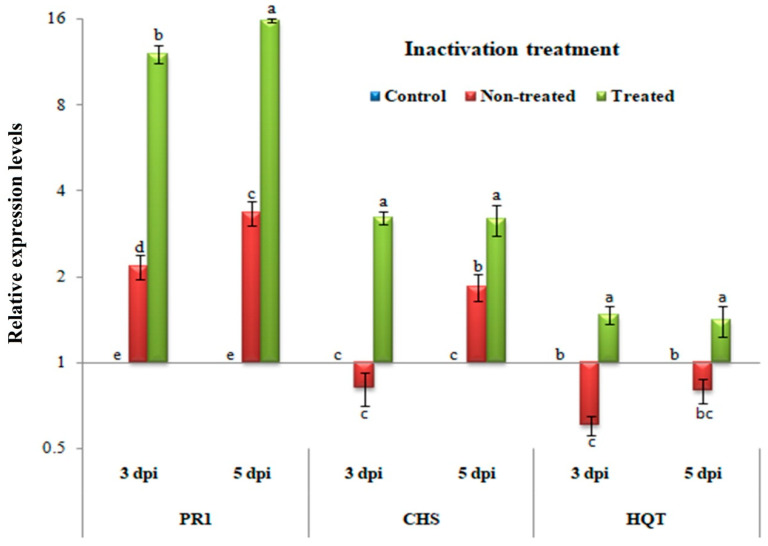
A histogram showing the relative expression levels of the PR-1, CHS, and HQT genes at 3 and 5 dpi of *H. tuberculatum* WPE treatments (200 μg/mL) in the inactivity assay. Control = mock-treated plants; non-treated = plants inoculated with TMV only without any treatment; treated = plants treated with *H. tuberculatum* WPE, 24 h before inoculation of TMV for the protective assay and 24 h after inoculation of TMV for the inactivity assay. Columns represent a mean value from three biological replicates and bars indicate SD. Significant differences between samples were determined by one-way ANOVA using CoStat software. Means were separated by the LSD test at *p* ≤ 0.05 and indicated by lowercase letters. Columns with the same letter do not differ significantly.

In Figure 5, similarly to the protective treatment, significant increases in the relative expression levels of PR-1, CHS, and HQT were observed in plant tissues treated with *H. tuberculatum* WPE when compared to the control and non-treated plants (*p* ≤ 0.05) at 3 and 5 dpi. Compared to the mock tissues, a significant up-regulation of PR-1 with relative expression levels of 2.162- and 3.342-fold change was observed in non-treated tissues at 3 and 5 dpi, respectively. However, *H. tuberculatum* WPE-treated tissues showed an increase in the transcription of PR-1 with relative expression levels of 12.036- and 15.763-fold change at 3 and 5 dpi, respectively, compared to the controls. Regarding the CHS gene, down-regulation with a relative expression level of 0.815-fold change was observed in non-treated tissues at 3 dpi. In contrast, *H. tuberculatum* WPE-treated tissues showed up-regulation with a significant relative expression level of 1.848-fold change at the same time when compared to the control tissues. Subsequently, at 5 dpi, an increase in the expression with relative expression levels of 3.215- and 3.172-fold change was observed in non-treated and *H. tuberculatum* WPE-treated tissues, respectively. For the HQT transcripts, significant up-regulation with relative transcriptional levels of 1.470- and 1.401-fold change were found only in *H. tuberculatum* WPE-treated tissues at 3 and 5 dpi, respectively, when compared to the controls. Conversely, the non-treated tissues exhibited down-regulation of HQT with relative transcriptional levels of 0.603- and 0.795-fold change lower than the controls at 3 and 5 dpi, respectively.

## 4. Discussion

Several polyphenolic compounds from the ethanolic WPE of *H. tuberculatum* were identified by HPLC, such as resveratrol, kaempferol, myricetin, rutin, quercetin, rosmarinic acid, catechol, *p*-hydroxybenzoic acid, and benzoic acid. The *H. tuberculatum* WPE showed the presence of total phenol content (TPC) ranging between 0.27 and 11.97 mg gallic acid equivalent (GAE)/g dry matter and a whole flavonoid content from 0.05 to 1.50 mg equivalent of rutin/g of dry matter [66]. The TPC was 46.2 mg GA/g sample, and the main chemical constituents of quercetin derivatives, cinnamic acid, ferulic acid, vanillic acid, and benzoic acid were found in the ethanol extract of the aerial parts of *H. tuberculatum* [67]. The TPC was observed to be 561.22 mg/g of GAE and the flavonoids 165.54 mg/g of quercetin equivalent [68]. The ethyl acetate extract of *H. tuberculatum* leaves was the most abundant extract in phenolics and flavonoids, with 262 mg GAE/g and 99.1 mg quercetin equivalent/g of dry weight, respectively [42].

In the present study, all of the examined *H. tuberculatum* WPE concentrations exhibited antifungal properties against the linear growth of two fungal isolates compared with the positive control (fluconazole), which commercially used in a rapid susceptibility testing useful method to determine the optimal treatment for infection with resistant isolates [69].

Many strategies have been used to reduce agricultural losses caused by fungal diseases including spraying of chemicals, biological control [70], and azoles fungicide [71]. The azoles group gave high minimal inhibitory concentrations (MICs) against the most *Fusarium* species [72]. *Candida albicans* is usually acutely susceptible to fluconazole; fluconazole MICs for approximately 90% of *C. albicans* isolates are ≤1 μg/mL [73]. Some non-*C. albicans* yeasts have been noted to have decreased susceptibility or resistance to fluconazole [73].

Our recent research similarly showed the highest inhibition of *R. solani*, *B. cinerea*, and *F. culmorum* growth by 64.4%, 100%, and 38.5%, respectively, with the ethanol extract of *Coccoloba uvifera* L. at 3% [48]. The *Eucalyptus camaldulensis* L. aerial parts n-hexane extract showed the same strong fungicidal property against the two fungal isolates, *F. culmorum* and *R. solani* especially at the concentration of 3% [50]. In the same way, wood samples treated with *Acacia saligna* water extract showed inhibition of fungal mycelial growth of *F. culmorum* and *R. solani* [15]. In a study performed by Sabry et al. [74] the ethanolic extract of the aerial parts of *H. tuberculatum* demonstrated an efficient antifungal property against *Aspergillus fumigates*, *Geotricum candidum* and *Syncephalastrum racemosum* with (MIC 0.49, 0.12, and 1.95 µg/mL). While the antimicrobial tests of *H. tuberculatum* extracts were more effective against Gram-negative bacteria than Gram positive ones. The best antibacterial activity was exhibited by methanolic extract, which was also active against *C. albicans* [75].

Other works reported the cytotoxicity of the extracted parts of *H. tuberculatum* on other pests, and the hexane, chloroform, ethyl acetate, butanol, methanol, and water extracts of the leaves of *H. tuberculatum* displayed significant cytotoxic activity against brine shrimp larvae. At the same time, the ethanol extract of the aerial parts of *H. tuberculatum* has shown good insecticidal activity against *Culex quinquefasciatus* [38]. In comparison, the oil of *H. tuberculatum* has been observed to have a slightly antimicrobial effect on the growth of *Escherichia coli*, *Salmonella choleraesuis*, and *Bacillus subtilis*, as well as antifungal activity against *C. lunata* and *F. oxysporum* growth. Still, it does not affect the germination of their spores [40]. In a different way the fungicidal property of the *H. tuberculatum* might be went to its composition of flavonoids, tannins, phenolic acids, especially resveratrol, which displays better antifungal than antibacterial activity, as demonstrated by the minimum inhibitory concentrations (MICs). For the fungal species *C. albicans*, *Saccharomyces cerevisiae* and *Trichosporon beigelii*, the inhibitory activity is 10–20 μg/mL [76]. Resveratrol displays inhibitory activity against the plant pathogen *B. cinerea*, the causal agent of grey mold, where reduced germination of *B. cinerea* conidia and mycelial growth is observed at concentrations of 60–140 μg/mL [77]. While for the antibacterial activity the resveratrol exhibited MIC > 400 against the Gram negative bacteria, *Escherichia coli*, *Salmonella enterica* serovar Typhimurium, and *Pseudomonas aeruginosa* [78]. In our study, anti-TMV, protective, and inactivating, the activity of *H. tuberculatum* WPE on *C. amaranticolor* tissues using the half-leaf method [53] was investigated for the first time. Mainly, the inhibitory effects, accumulation levels of TMV CP, and relative expression levels of three defense-related genes (i.e., PR-1, CHS, and HQT) at 3 and 5 dpi were evaluated. Overall, our results indicated that *H. tuberculatum* WPE had an inhibitory effect against TMV infection. In the current study, the application of *H. tuberculatum* WPE (200 µg/mL) showed a significant reduction in local lesion symptoms when *C. amaranticolor* tissues were treated either 24 h before or with to viral challenge. The inactivity of *H. tuberculatum* WPE exhibited a strong inhibitory effect (approximately 96%), while the protective activity showed an inhibitory effect of 65%. The treatment of TMV with the aqueous extract of *Bryophyllum daigremontianum* (200 mg/mL) before mechanical inoculation significantly reduced the number of local lesions in *N. tabacum* var. *Xanthi*, *N. glutinosa*, and *V. faba* plants and showed inhibitory effects ranging from 51.45% to 86.08% [79]. The qPCR results confirmed the antiviral activity of *H. tuberculatum* WPE against TMV infection, which resulted in a considerable decrease in the viral accumulation level inside the treated tissues. The relative accumulation levels of TMV CP in *C. amaranticolor* tissues were 3.866- and 2.470-fold change in the protective activity and inactivity treatments, respectively, while non-treated tissues exhibited 28.918- and 27.042-fold change, respectively, at 5 dpi. These results suggest that *H. tuberculatum* WPE can directly inactivate TMV and may interfere with coat proteins or may inhibit viral replication inside plant cells. Jing et al. [31] reported that several plant extracts inhibited TMV infection through preventing the infection or spread of TMV, as well as the inhibition of viral replication.

In general, the direct and indirect inhibition of viral replication, through simultaneous activation of the host’s innate immune system and by inducing SAR against viral infection, are two mechanisms of antiviral agents [31,37]. Regarding the stimulating effect on *C. amaranticolor* tissues, *H. tuberculatum* WPE induced and activated the expression of three defense-related genes (i.e., PR-1, CHS, and HQT).

PR-1 is considered a principal regulator of SAR and could be a marker of plant early defense responses [80]. Moreover, salicylic acid (SA) is a vital signal phytohormone molecule of SAR in plants [81], and its role in plant immunity has been known for over two decades. The activation of SA in response to pathogens is associated with the accumulation and expression of PR-1 as a SA marker gene [59]. In the present study, the non-treated *C. amaranticolor* tissues challenged with TMV showed induction of PR-1 with relative expression levels of 1.926- and 7.467-fold change and 2.162- and 3.342-fold change in protective activity and inactivity treatments at 3 and 5 dpi, respectively. However, the *H. tuberculatum* WPE-treated tissues exhibited overexpression of PR-1 with transcriptional levels of 12.436- and 12.036-fold change in protective activity and inactivity treatments, respectively, at 3 dpi. At 5 dpi, PR-1 continued to accumulate, reaching maximum levels of 14.750- and 15.763-fold change in protective activity and inactivity treatments, respectively, when compared to the controls. Consequently, we suggest that *H. tuberculatum* WPE may contain elicitor molecules that activate the immune defense system besides the inhibition of TMV replication. In this context, tobacco plants treated with *Sophora flavescens*, *Forsythia suspense*, and *Lonicera japonic* extracts exhibiting the induction and up-regulation of PR-1 resulted in the development of SAR against TMV [82].

Besides, as the first enzyme in the flavonoid pathway that catalyzes the synthesis of naringenin chalcones, CHS is strictly required in various plant tissues for flavonoid production [59,83]. Compared to mock tissues at 3 dpi, the CHS transcripts were induced only in *H. tuberculatum* WPE-treated tissues with relative expression levels of 1.778- and 3.215-fold change for protective activity and inactivity treatments, respectively. At 5 dpi, up-regulation of CHS in non-treated tissues was observed, while *H. tuberculatum* WPE-treated tissues exhibited an increase in the transcriptional levels of CHS. The down-regulation of CHS at 3 dpi of non-treated tissues suggests that TMV infection suppresses naringenin chalcones biosynthesis in early infection. Interestingly, the application of *H. tuberculatum* WPE in the protective activity and inactivity assays showed the highest induction of CHS that is strictly required for flavonoid production naringenin chalcones, which are considered the primary precursors and constitute the main intermediates for the synthesis of many flavonoids by the action other enzyme sets [8,84].

Chlorogenic acid (CGA), one of the most polyphenolic compounds, plays important roles in increasing plant resistance and in inhibiting pathogens, including viruses [85,86,87]. HQT is the key enzyme in the biosynthesis of CGA, while it catalyzes caffeoyl-CoA and quinic acid to form CGA [88]. In the present study, the transcription of CHS was wholly suppressed and down-regulated in non-treated tissues at 3 and 5 dpi of the protective activity and inactivity treatments when compared to the controls. The overexpression of HQT was associated with increases in chlorogenic acid content and versa [88]. Consequently, TMV was able to suppress chlorogenic acid biosynthesis inside infected tissues.

On the other hand, the application of *H. tuberculatum* WPE induced HQT transcripts in both treatments, i.e., protective activity and inactivity, at 3 and 5 dpi. A higher expression level of HQT (1.573-fold change) was shown in *H. tuberculatum* WPE-treated tissues of the protective activity assay at 5 dpi. In comparison, a high expression level of HQT in the inactivity assay (1.470-fold change) was observed at 3 dpi. Based on the current results, *H. tuberculatum* WPE induced and activated HQT transcripts that correlated with increasing CGA accumulation inside treated tissues.

## 5. Conclusions

We firstly examined *H. tuberculatum* WPE as a novel antiviral agent against plant viruses, and our results suggest that it contains compounds that penetrate plant cells, play significant roles in SAR, inhibit infection, and directly inactivate TMV. Consequently, *H. tuberculatum* WPE may be considered as a promising source of both antifungal and antiviral substances for practical use and for developing plant-derived compounds for the effective management of plant diseases.

## Figures and Tables

**Table 1:** Nucleotide sequences of the qRT-PCR primers used in this study.

Primer Name	Abbreviation	Direction	Sequence (5′–3′)	References
Pathogenesis-related protein-1	*PR-1*	Forward	CCAAGACTATCTTGCGGTTC	[61]
		Reverse	GAACCTAAGCCACGATACCA	
Chalcone synthase	*CHS*	Forward	CACCGTGGAGGAGTATCGTAAGGC	[62]
		Reverse	TGATCAACACAGTTGGAAGGCG	
Hydroxycinnamoyl Co A quinate hydroxycinnamoyl transferase	*HQT*	Forward	CCCAATGGCTGGAAGATTAGCTA	[62]
		Reverse	CATGAATCACTTTCAGCCTCAACAA	
Beta-actin	*β-actin*	Forward	ATGCCATTCTCCGTCTTGACTTG	[63]
		Reverse	GAGTTGTATGTAGTCTCGTGGATT	
Tobacco mosaic virus-coat protein	*TMV-CP*	Forward	ACGACTGCCGAAACGTTAGA	[64]
		Reverse	CAAGTTGCAGGACCAGAGGT	

**Table 2:** Polyphenolic compounds identified in the ethanol extract of the *Haplophyllum tuberculatum* whole plant by High Performance Liquid Chromatography (HPLC).

Compound	Amount (mg/kg)
Chlorogenic acid	ND
*p*-Coumaric acid	ND
Naringenin	ND
Pyrogallol	ND
Gallic acid	8.35
Ferulic acid	26.86
Catechin	27.43
Quinol	33.85
Syringic acid	35.91
Caffeic acid	39.63
Vanillic acid	45.14
Ellagic acid	45.39
Cinnamic acid	46.79
*o*-Coumaric acid	81.22
Catechol	120.66
Benzoic acid	199.51
*p*-Hydroxy benzoic acid	221.47
Rosmarinic acid	387.33
Quercetin	401.04
Rutin	487.04
Myricetin	561.18
kaempferol	1735.23
Resveratrol	5178.58

ND, not detected.

**Table 3:** Antifungal property of wood treated with *H. tuberculatum* WPE against the growth of *F. culmorum*, and *R. solani*.

Treatment	Inhibition Percentage of Fungal Linear Growth (%)	
	*F. culmorum*	*R. solani*
*H. tuberculatum* WPE (1%)	46.29d ± 0.37 *	49.62c ± 0.37
*H. tuberculatum* WPE (2%)	72.96b ± 0.37	66.29b ± 0.37
*H. tuberculatum* WPE (3%)	82.96a ± 0.37	93.70a ± 0.37
Fluconazole (25 µg)	53.70c ± 0.37	42.96d ± 0.37
Control (DMSO 10%)	0.00e	0.00e
LSD 0.05	1.04	1.04

* Values are reported as means ± standard error (SE). Means with the letter within the same column are not significantly difference according to LSD0.05. DMSO: Dimethyl sulfoxide.
